# Landscape management and domestication of *Stenocereus pruinosus* (Cactaceae) in the Tehuacán Valley: human guided selection and gene flow

**DOI:** 10.1186/1746-4269-8-32

**Published:** 2012-08-14

**Authors:** Fabiola Parra, José Juan Blancas, Alejandro Casas

**Affiliations:** 1 Centro de Investigaciones en Ecosistemas (CIECO), Universidad Nacional Autónoma de México (campus Morelia), Antigua Carretera a Pátzcuaro 8711 Col. Ex Hacienda de San José de la Huerta, Morelia, Michoacán, 58190, Mexico

**Keywords:** Artificial selection, Columnar cacti, Domestication, Genetic resources conservation, Landscape management, Morphological variation, *Stenocereus pruinosus*

## Abstract

**Background:**

Use of plant resources and ecosystems practiced by indigenous peoples of Mesoamerica commonly involves domestication of plant populations and landscapes. Our study analyzed interactions of coexisting wild and managed populations of the pitaya *Stenocereus pruinosus*, a columnar cactus used for its edible fruit occurring in natural forests, silviculturally managed in milpa agroforestry systems, and agriculturally managed in homegardens of the Tehuacán Valley, Mexico. We aimed at analyzing criteria of artificial selection and their consequences on phenotypic diversity and differentiation, as well as documenting management of propagules at landscape level and their possible contribution to gene flow among populations.

**Methods:**

Semi-structured interviews were conducted to 83 households of the region to document perception of variation, criteria of artificial selection, and patterns of moving propagules among wild and managed populations. Morphological variation of trees from nine wild, silviculturally and agriculturally managed populations was analyzed for 37 characters through univariate and multivariate statistical methods. In addition, indexes of morphological diversity (MD) per population and phenotypic differentiation (PD) among populations were calculated using character states and frequencies.

**Results:**

People recognized 15 pitaya varieties based on their pulp color, fruit size, form, flavor, and thorniness. On average, in wild populations we recorded one variety per population, in silviculturally managed populations 1.58 ± 0.77 varieties per parcel, and in agriculturally managed populations 2.19 ± 1.12 varieties per homegarden. Farmers select in favor of sweet flavor (71% of households interviewed) and pulp color (46%) mainly red, orange and yellow. Artificial selection is practiced in homegardens and 65% of people interviewed also do it in agroforestry systems. People obtain fruit and branches from different population types and move propagules from one another. Multivariate analyses showed morphological differentiation of wild and agriculturally managed populations, mainly due to differences in reproductive characters; however, the phenotypic differentiation indexes were relatively low among all populations studied. Morphological diversity of *S. pruinosus* (average MD = 0.600) is higher than in other columnar cacti species previously analyzed.

**Conclusions:**

Artificial selection in favor of high quality fruit promotes morphological variation and divergence because of the continual replacement of plant material propagated and introduction of propagules from other villages and regions. This process is counteracted by high gene flow influenced by natural factors (pollinators and seed dispersers) but also by human management (movement of propagules among populations), all of which determines relatively low phenotypic differentiation among populations. Conservation of genetic resources of *S. pruinosus* should be based on the traditional forms of germplasm management by local people.

## Background

Studies of subsistence patterns among indigenous cultures of Mesoamerica have documented that people commonly manage their territories for agriculture, animal raising, and use of forest products which provide complementary resources to satisfy households’ needs. Such subsistence pattern has been called by Toledo and collaborators [1-3] the multiple using of natural resources and ecosystems, and involves use of both components and processes of natural and artificial ecosystems. In such context, particularly relevant are traditional agroforestry systems, which include components of natural vegetation managed through silvicultural practices and domesticated or semi-domesticated components managed through agricultural practices [2-4]. Agroforestry, silvo-pastoral areas, and forest management systems are all interconnected in territories, and processes occurring in one influence those occurring in the others.

Agroforestry systems include a high diversity of production systems; among them, particularly important in rural areas of Mexico are homegardens and the traditional multi-crop fields called “milpa” managed apart from houses. These systems and the surrounding landscape are widely recognized for their high capacity of conservation of natural biodiversity [4-7] and agrobiodiversity [7-9]. And such capacity has been documented in tropical wet areas [6,9] as well as in temperate, arid, and semi-arid zones [3,10-12]. Also, it has been documented that local peoples obtain from these systems a variety of products for complementing their subsistence needs [4,6].

All the managed systems mentioned may involve domestication, an evolutionary process guided by artificial selection *sensu* Darwin [13], determining morphological, physiological and genetic divergences among organisms driven by human purposes. Recently, several authors have recognized that genetic drift and gene flow may also be driven by humans and that these processes are relevant for analyzing domestication [14-16]. Domestication has commonly been analyzed in plants associated to agriculture and more scarcely in plants under silvicultural management, but recent studies throughout the world reveal that this process is more common than previously considered [17-26]. Most studies of domestication have analyzed the process occurring at biological populations’ level, but it may also occur at landscape level by modeling both physical and biotic components of territories, as well as their interrelationships and processes in order to satisfy human needs. An integrated approach of analyzing domestication at both population and landscape levels may allow a better understanding of interactions of the processes in both agricultural and non-agricultural systems. Also, such approach may allow analyzing domestication operating on particular species within the context of general management strategies of landscapes and particular resources.

Agricultural and non-agricultural landscapes in territories are dynamic evolving socio-ecological systems [27]. In regions that are centers of origin of domestication, native varieties of crops have coexisted with their wild relatives and human cultures managing them [2], and such coexistence is a main factor favoring generation of agrobiodiversity. Therefore, strategies for conserving native agrobiodiversity need considering maintenance of biological sources of diversity, as well as human cultural motives that generate divergence [28]. For agrobiodiversity conservation, identifying and conserving populations of crop wild relatives and identification of interesting alleles for future breeding efforts is of high priority in order to ensure occurrence of gene flow among wild and domesticated populations [28-30]. For maintaining human cultural motives generating diversity, it is crucial favoring diversified use of crops, interchange of varieties, knowledge and management techniques [28]. Agroforestry systems are important bridges of gene flow among components of a matrix of landscapes [4,31], as well as reservoirs of traditional knowledge, plant management and processes of domestication [3-5,17,18,32,33]. Therefore, these systems are crucial for bio-cultural conservation agendas.

Mesoamerica is one of the areas with higher biological and human cultural diversity [34-36] and one of the main centers of domestication of plants of the World [37-39]. From nearly 7,000 plant species used by the Mesoamerican cultures, Caballero *et al.*[40] identified about 700 native plant species under incipient management and nearly 150 native species domesticated and managed intensively in agricultural systems. Therefore, Mesoamerica is an important area for studying how on-going mechanisms of domestication do operate [16].

In order to analyze processes of artificial selection operating at landscape level, we studied the case of pitaya, the columnar cactus *Stenocereus pruinosus* which can be found in wild, silviculturally and agriculturally managed populations in territories of human communities of the Tehuacán Valley, Central Mexico. Wild populations are groups of pitaya plants forming part of natural vegetation, reproducing and growing independently of humans; silviculturally managed populations are groups of plants originally wild but deliberately let standing, promoted, and cared in areas transformed for the multi-crop milpa agroforestry systems; and agriculturally managed populations are stands of plants propagated and cared in homegardens. Fruits of this cactus species are edible and their cultural value can be appreciated in relation to their intensive commercialization at both communitarian and regional traditional markets. In the Tehuacán Valley, *S. pruinosus* can be found wild as part of tropical deciduous forests associated to alluvial valleys of seasonal rivers [16]. It also forms part of agroforestry systems cultivating the multicrop milpa in transformed forests of columnar cacti such as “chichipera forest” dominated by *Polaskia chichipe*[4,41], “garambullal forest” dominated by *Myrtillocactus schenkii*[3,4], and “jiotillales”, dominated by *Escontria chiotilla*[4,16,42]. In these systems individual plants of *S. pruinosus* and other species of columnar cacti are let standing when crop fields are open, but in addition people use to plant vegetative propagules from wild and agriculturally managed populations into homegardens [16]. Agriculturally managed populations are formed by plants cultivated in homegardens, which are principal areas of artificial selection of a number of plant species [3,16].

In a previous study [16] we documented that management by local people determines morphological and genetic divergences between wild and managed populations of *S. pruinosus*. Such divergences are caused by artificial selection favoring plants producing larger and sweeter fruits with pulp colors more diverse than the red pulp predominant in wild fruits, as well as fruit peel thicker or thinner than that characterizing fruits of wild plants, among other features. However, in our previous studies we also found that there are high levels of gene flow among all these population types. Gene flow is associated to movement of pollen and seeds by natural agents (mainly bats and birds, respectively), and we have supposed that movement of vegetative propagules by humans may also be relevant. Gene flow continually counteract processes of divergence determined by both natural and artificial selection and contributes to maintain and in some cases even increase genetic diversity in managed populations [16,42].

This study aimed at analyzing criteria and mechanisms of artificial selection and their consequences on morphological diversity and phenotypic divergences among wild and managed populations. Similarly to patterns documented for *Stenocereus stellatus*[3,32] and according to our previous population genetics’ studies in *S. pruinosus*[16,42], we hypothesized that managed populations would have higher morphological diversity than wild populations and that phenotypic divergence between wild and agriculturally managed populations would be higher than that between wild and silviculturally managed populations. Management and artificial selection of *S. pruinosus* is relatively more intense than that occurring in *S. stellatus*[16,42]; therefore, we expected that these trends in *S. pruinosus* would be more marked than in *S. stellatus*. In addition, we aimed at understanding human mechanisms determining spatial movement of propagules among wild and managed populations coexisting in a territory with a mosaic of wild and managed environmental units. In this respect, we hypothesized that along with natural mechanisms influenced by pollinators and seed dispersers, deliberate interchange of sexual and vegetative propagules favored by people could contribute to explain the high levels of gene flow documented previously.

## Methods

### Study area

**Figure 1:**
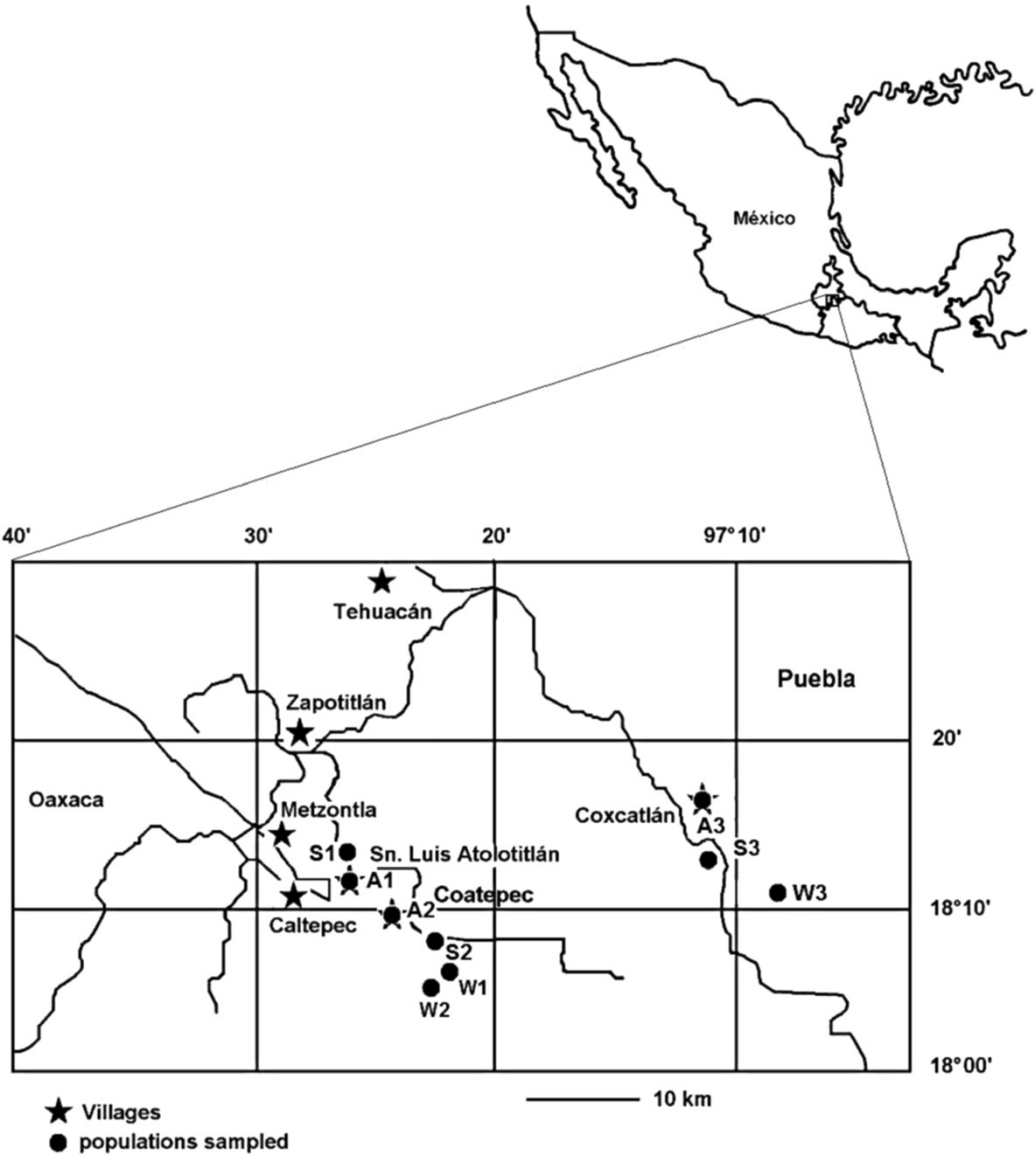
**Study area.** The Tehuacán Valley. Location of the villages and populations of *Stenocereus pruinosus* studied. W1 (Wild Santa Lucía); W2 (Wild Fiscal), W3 (Wild Coxcatlán), S1 (Silviculturally Managed San Luis Atolotitlán), S2 (Silviculturally Managed Coatepec) S3 (Silviculturally Managed Coxcatlán), A1 (Agriculturally managed San Luis Atolotitlán), A2 (Agriculturally managed Coatepec), A3 (Agriculturally managed Coxcatlán).

Our study was conducted in territories of the villages of San Luis Atolotitlán, Coatepec, and Coxcatlán, in the Tehuacán Valley, central Mexico (Figure 1). Three wild, three silviculturally managed and three agriculturally managed populations were studied, sampling 30 trees per wild and agriculturally managed population, but samples in silviculturally managed populations varied from15 to 30 plants according to their availability in agroforestry systems. Wild populations are located in the sites Santa Lucía and Fiscal within the territory of Coatepec, and in the site Cueva del Maíz (Maize Cave) within the territory of Coxcatlán, as part of natural patches of tropical deciduous forest associated to alluvial valleys of seasonal rivers (Figure 1). In these habitats the columnar cacti *Pachycereus weberi*, *P. hollianus*, *Escontria chiotilla*, *Stenocereus pruinosus*, and *S. stellatus*, are co-dominant with the trees *Prosopis laevigata* (Leguminosae), *Cyrtocarpa procera* (Anacardiaceae), *Ceiba aesculifolia* (Malvaceae), *Bursera morelensis* (Burseraceae), and *Parkinsonia praecox* (Caesalpinaceae). Silviculturally managed populations are located in scattered areas of milpa agroforestry systems used for seasonal agriculture of maize near the villages of San Luis Atolotitlán, Coatepec, and Coxcatlán. In this system people let standing individuals with useful phenotypes when natural vegetation is cleared for agriculture [43]. Finally, the agriculturally managed populations are in homegardens of the villages mentioned.

### Ethnobotanical studies

After previous assemblies and permission by local authorities, we conducted ethnobotanical studies through semi-structured interviews to women and men of 63 households of the villages of San Luis Atolotitlán, Coatepec, and Coxcatlán, Puebla. Households were selected at random in San Luis Atolotitlán and Coatepec, where practically all homegardens have *S. pruinosus* among their components; but in Coxcatlán we selected the households interviewed according to presence of *S. pruinosus* in their homegardens. Interviews were directed to document perception of variation, nomenclature and classification of traditional varieties, criteria of artificial selection of *S. pruinosus* by local people, and how these criteria operate in different management conditions. Interviews were also directed to document how people select and carry out movement of vegetative propagules among populations coexisting within a territory, and what people do with seedlings and juvenile plants that become naturally established without human interference in parcels and other managed areas. Additional 20 semi-structured interviews were conducted with people of San Luis Atolotitlán and Coatepec who manage agroforestry systems where *S. pruinosus* occur, in order to document how agroforestry systems are connected with forests and homegardens and the importance of the multiple ways of plant and landscape management for conserving local biodiversity. Ethnobotanical information was analyzed through descriptive statistics.

### Patterns of morphological variation

A total of 37 morphological characters (Table 1) were measured (three to five measurements per character per tree sampled) in samples of 15 to 30 cactus trees in each of the populations studied. Patterns of morphological similarity and differentiation among individual plants within populations and among populations were analyzed through multivariate statistical methods. We used Principal Components Analysis (PCA), Discriminant Function Analysis (DFA) and Cluster Analysis (CA) [44] to classify the individual plants sampled according to their morphological similarity, in this way exploring whether similarities are related to their management type.

**Table 1:** **Mean values ± s. e. of morphological characters analyzed in wild, silviculturally and agriculturally managed populations of *S. pruinosus ***

**Character**	**Wild populations**	**Silviculturally managed populations**	**Agriculturally managed populations**	**PC1**	**PC2**
Fruit Volume (gr)**	B	B	A	−0.868	−0.182
	56.577 ±3.241	67.048 ±4.188	127.882 ±4.909		
Total fruit weight (g)**	B	B	A	−0.880	−0.205
	55.913 ±3.137	67.369 ±3.983	127.109 ±4.634		
Pulp weight (gr)**	B	B	A	−0.851	−0.224
	30.881 ±2.019	36.447 ±2.505	80.064 ±3.553		
Peel weight (gr)**	C	B	A	−0.783	−0.095
	24.977 ±1.473	30.588 ±1.733	46.413 ±1.437		
Peel thickness (cm)*	A	AB	B	−0.031	0.020
	0.365 ±0.014	0.342 ±0.011	0.324 ±0.011		
Number of areoles per peel cm^2^**	A	A	B	0.655	0.174
	2.007 ±0.048	2.017 ±0.034	1.672 ±0.045		
Number of seed per fruit**	B	B	A	−0.480	−0.100
	1212.279 ±52.420	1176.854 ±50.687	1597.572 ±59.397		
seed weight (gr)**	B	B	A	−0.322	0.228
	0.00209 ±0.00001	0.00208 ±0.00001	0.00224 ±0.00001		
Sugar content in pulp (Brix)**	B	A	A	−0.318	−0.090
	6.720 ±0.251	8.140 ±0.281	8.643 ±0.220		
Pulp acidity (pH)**	B	B	A	−0.283	−0.259
	4.302 ±0.063	4.314 ±0.066	4.593 ±0.060		
Pulp color	A	A	A	0.177	0.0015
	1.78 ±0.166	1.89 ±0.165	2.16 ±0.172		
Number of stem ribs**	B	A	A	0.082	0.324
	5.751 ±0.039	5.971 ±0.039	5.938 ±0.048		
Stem rib width (cm)	A	A	A	−0.265	0.517
	3.783 ±0.069	3.834 ±0.095	3.694 ±0.067		
Stem rib depth (cm)*	B	B	A	−0.325	0.208
	3.778 ±0.039	3.851 ±0.048	4.118 ±0.106		
Distance among rib areoles (cm)	A	A	A	−0.179	0.183
	3.167 ±0.052	3.268 ± 0.0629	3.277 ± 0.061		
Spines/areole	B	A	A	−0.060	−0.006
	8.029 ±0.132	8.933 ± 0.137	9.185 ± 0.132		
Size of central spines (cm)**	B	A	C	0.294	−0.076
	2.630 ±0.094	3.081 ±0.135	1.914 ±0.068		
Branch width (cm)**	C	B	A	−0.416	0.422
	10.403 ±0.099	11.119 ±0.150	11.823 ±0.113		
Height (m)*	A	B	AB	0.043	0.727
	4.751 ±0.133	4.176 ±0.139	4.385 ±0.172		
Plant size (m3)	A	A	A	−0.015	0.736
	14.328 ±1.620	13.451 ±1.924	13.525 ±1.612		
Branch number**	B	B	A	−0.224	0.603
	33.622 ±3.641	33.595 ±3.040	58.611 ±6.475		
Flower tube length (cm)**	C	B	A	−0.710	−0.176
	9.240 ±0.136	9.675 ±0.132	10.317 ±0.102		
Corola maximun diameter (cm)**	C	B	A	−0.407	0.143
	5.586 ±0.119	6.000 ±0.113	6.474 ±0.127		
Corola maximun intern opening (cm)	A 3.008 ±0.049	A 3.067 ±0.048	A 3.170 ±0.046	−0.302	0.357
Flower tube minimun diameter(cm)**	C	B	A	−0.632	0.239
	1.059 ±0.013	1.116 ±0.015	1.188 ±0.013		
Pericarpel diameter (cm)**	B	B	A	−0.786	0.120
	1.461 ± 0.024	1.520 ± 0.024	1.655 ± 0.020		
Pericarpel length (cm)**	B	B	A	−0.806	−0.135
	2.117 ±0.046	2.168 ±0.046	2.558 ±0.047		
Nectar chamber length (cm)**	B	B	A	−0.473	−0.352
	1.616 ±0.029	1.644 ±0.028	1.796 ±0.033		
Nectar chamber diameter (cm)	A	A	A	−0.254	0.466
	0.867 ± 0.021	0.851 ± 0.016	0.860 ± 0.013		
Ovary length (cm)**	B	B	A	−0.795	−0.043
	1.057 ±0.027	1.079 ±0.035	1.341 ±0.025		
Ovary diameter (cm)*	B	AB	A	−0.618	0.358
	0.706 ± 0.020	0.717 ± 0.175	0.767 ± 0.010		
Stile lengtht (cm)**	B	A	A	−0.111	−0.263
	4.804 ±0.073	5.168 ±0.086	5.121 ±0.058		
Maximum stigma length (cm)**	B	B	A	−0.604	−0.074
	1.205 ±0.028	1.248 ±0.036	1.377 ±0.030		
Number of stigma lobes**	B	A	A	−0.268	0.326
	8.716 ±0.149	9.382 ±0.189	9.357 ±0.163		
Average stigma length (various lobes) (cm)**	C	B	A	−0.563	−0.138
	1.010 ±0.036	1.154 ±0.034	1.277 ±0.030		
Anters length (cm)**	B	B	A	−0.299	0.056
	0.257 ±0.004	0.263 ±0.004	0.283 ±0.003		
Anters width (cm)	A	A	A	−0.144	0.018
	0.092 ± 0.002	0.091 ± 0.001	0.093 ±0.001		

Different capital letters among populations indicate significant differences according to ANOVA and Tukey tests (*p ≤ 0.05, **p ≤ 0.01). The last columns show eigenvectors of the first (PC1) and second (PC2) principal components according to PCA.

Multivariate analyses PCA and DFA were performed using a data matrix with morphological characters considered as variables and individual trees sampled considered as operative taxonomic units (OTUs). CA was performed considering populations as OTUs. Due to differences associated to character type and measurement units, we standardized the data matrix using the algorithm Y_0_ = (Y-a)/b; where Y_0_ is the standardized value, Y is the real value of character state, a is its average and b its standard deviation [44]. PCA and CA were performed with NTSys 2.02 [45], and DFA using IBM SPSS Statistics 19. CA based on a similarity matrix calculated using the Pearson Correlation Coefficient, clustering by the technique of unweighted arithmetic average (UPGMA). In order to validate the CA we calculated a cophenetic correlation matrix and the *r* value [44]. PCA was performed based on a similarity matrix using the coefficient of variance-covariance. Eigen vectors allowed identifying morphological characters with higher meaning to classify morphological patterns. DFA included a multiple analysis of variance (MANOVA) for testing significance of differences among wild, silviculturally and agriculturally managed populations. Tukey tests were also performed to identify the type of management showing significant differences. These tests were performed to identify trends of variation according to management and artificial selection intensity.

One-way ANOVA and Tukey (95% confidence) multiple-range tests were performed through IBM SPSS Statistics 19 to identify how morphological characters studied differed among populations according to their management type.

### Morphological diversity and phenotypic differentiation

An index of morphological diversity (MD) was calculated based on the Simpson diversity index following methods for estimating morphological diversity proposed by Casas *et al.*[32] and Blancas *et al.*[46] to summarize information on the amount of variation of all variables. This index was defined as MD = 1-Σ_1-s_(p_i_)^2^ in which p_i_ is the proportion of the total number of individual plants sampled in a population showing the _i_th state of morphological character and s is the number of states of that character [32]. Frequencies of character states were first calculated with previous conversion to qualitative states, which were established based on intervals of values significantly different, according to one-way ANOVA comparing each character among populations studied and Tukey multiple range tests [32,47]. Significance of differences in MD among populations pooled by management type was tested with non parametric Wilcoxon tests (JMP, SAS Institute 1996). Phenotypic differentiation (PD) between pools of wild, silviculturaly, and agriculturally managed populations was analyzed using the algorithm of Nei’s genetic distance [48], which, considered the types and frequencies of morphological character states in populations as used by Blancas *et al.*[46]. According to this index, D = −ln*I*, where *I* = Σ = x_i_y_i_/(Σx_i_^2^Σy_i_^2^)^0.5^, x_i_ and y_i_ being frequencies of character states of different morphological features. Values of MD and PD of *S. pruinosus* populations were compared with those calculated for *S. stellatus, Polaskia chichipe, P. chende and M. schenckii* based on data by Blancas *et al.*[46], Casas *et al.*[18], and Cruz and Casas [49]. We additionally calculated these indexes for *Escontria chiotilla* based on morphometric data published by Arellano and Casas [50].

## Results

### Management of multiple ecological and cultural settings

Local people use to obtain fruits of *S. pruinosus* from forests as well as from agroforestry systems and homegardens. Cultivation of this species is mainly destined to consumption of fruit by households but commonly they also obtain incomes from their commercialization. All households interviewed affirmed to have commercialized this species fruit. Most of the households interviewed (65%) cultivate *S. pruinosus* at small scale (one to ten trees in their homegardens); nearly 25% of the households have more than ten trees (homegarden size being 500 ± 5 m^2^ on average) and an exceptional case was recorded managing several plantations (5000 ± 10 m^2^) with hundreds of trees mainly destined to fruit commercialization. In agroforestry systems *S. pruinosus* may be abundant; for instance, in San Luis Atolotitlán nearly 45% have more than 15 trees per parcel, but in Coatepec only 10% of households have more than 15 trees (parcel size being 1000 ± 100 m^2^ on average).

Gathering of fruit from wild populations complement requirements of fruits obtained in homegardens and agroforestry systems. It is practiced by nearly 70% of households interviewed. In Coatepec, nearly 90% of households gather fruits from wild populations from the sites Río Hondo and Fiscal, sampled in this study, which are 2 to 3 h away by foot path. People use to gather pitaya fruit while taking care of their goats.

In wild populations people commonly plant *in situ* branches of pitaya found in their walk, and take care of seedlings and juvenile plants. In some wild areas people used to establish seasonal small settlements with pitaya plantations. In these sites people collect fruits and commonly also branches for planting in homegardens.

Management of *S. pruinosus* in homegardens of the Tehuacán Valley is influenced by availability of plants in wild populations and agroforestry systems. In Coxcatlán, for instance according to local people, this cactus species is rare in homegardens since it is abundant in wild populations and agroforestry systems close to the town. There are few pitaya trees in homegardens, which were left standing when houses were constructed. Pitaya is abundant in the wild population of the Maize Cave (nearly 4 km), making unnecessary its cultivation, according to people. In Coatepec, for the contrary, wild populations of *S. pruinosus* are 15 to 20 km away from the town and according to people it is better to have trees of this species in their homegardens.

### Morphological variation and artificial selection

#### Classification of traditional varieties

In the villages studied in the Tehuacán Valley, a total of six main traditional varieties of pitaya are recognized according to their pulp color: red, yellow, orange, pink, purple and white. But each variety in turn may include two or more sub-varieties. In total, we recorded 15 names of pitaya traditional varieties. Characters used by people to classify traditional varieties are pulp color, fruit size (small and large) and form (spherical or ovoid), flavor (sweet and sour), and amount of spines on the fruit peel. The latter characters are used for qualifying sub-varieties of a variety determined by pulp color. For instance, high thorniness is used for naming the sub-varieties “pachona” or “china” of the orange pulp pitaya. Names of animals are used for designating traditional varieties according to their size; for instance, the varieties called “shicanela roja” (“red shicanela”, “shicanela” being the name of an ant species), the “amarilla hormiga” (“yellow ant”) and the “amarilla gorrioncito” (“yellow little sparrow”), are some names making reference to pitaya varieties producing small size fruit.

On average, every household has 2.19 ± 1.12 varieties of pitaya in their homegardens. Nearly 88% of households have 1 to 3 varieties, the most abundant being those of red pulp (occurring in 81% of homegardens sampled), followed by those of yellow pulp (in 56% of homegardens), and orange pulp (in 38% of homegardens sampled). Nearly 13% of households manage 4 to 5 varieties, more commonly in Coatepec (16% of households) and less commonly in San Luis Atolotitlán (8% of households). Varieties with pink and white pulp are scarcer than those with other pulp colors (Table 2).

**Table 2:** **Percentage of the households interviewed in villages of the Tehuacán Valley (n = 55 households) that manage different number of traditional varieties of *Stenocereus pruinosus ***

**Number of varieties**	**% of households**
1	31
2	31
3	20
4	9
5	4

In Coatepec the most common varieties are those of red, yellow and orange pulp, (which occur in 64%, 64%, 52% of the homegardens sampled, respectively) whereas in San Luis Atolotitlán the red pulp varieties are markedly abundant (in 96% of homegardens), followed by those of yellow pulp (in 50% of homegardens) and those of orange pulp (in 25% of homegardens). In Coxcatlán, varieties of red pulp are also the most abundant in all homegardens. Other varieties with yellow, orange and white pulp were found only in one homegarden which is a relatively large plantation.

In agroforestry systems we recorded on average 1.58 ± 0.77 varieties. Nearly 55% of parcels had one single variety, but 40% had two to three varieties, more in San Luis Atolotitlán than in Coatepec. In these systems some varieties are similar to those found in homegardens (see Table 3), the most common being variants of the red variety (58% of all agroforestry systems sampled), and those of the yellow variety (53% of all plots sampled). The white variety, rare in wild populations and homegardens (e.g. 8% of homegardens) was more abundant in agroforestry systems (21%).

**Table 3:** **Percentage of *S. pruinosus *varieties in different management systems (n = 55 homegardens and 20 agroforestry systems)**

**Variety**	**Wild populations**	**Agriculturally managed systems**	**Silviculturally managed systems**
Red	70	81	58
Yelllow	63	56	53
Orange	33	38	16
Purple	32	21	5
White	12	8	21
Pink	0	4	0

People interviewed said to have observed in the wild forests pitaya trees producing fruit of all pulp colors characterizing the main varieties. All interviewees agreed that the most common varieties are those of red pulp (70% of people interviewed) and those of yellow pulp (63% of interviewees). Variants of orange pulp were reported to have been observed in the wild by 33% of interviewees, those of purple pulp by 32%, and those of white pulp only by 12% of people interviewed.

#### Criteria of artificial selection and characters favored

**Figure 2:**
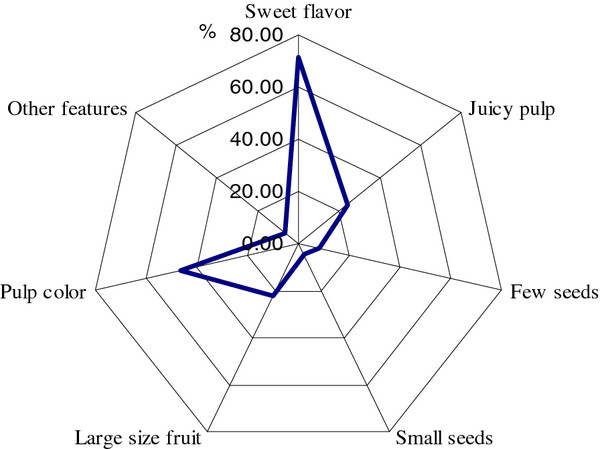
**Fruit characters preferred and selected by people interviewed.** Values in the plot represent percentage of people interviewed (n = 63).

**Figure 3:**
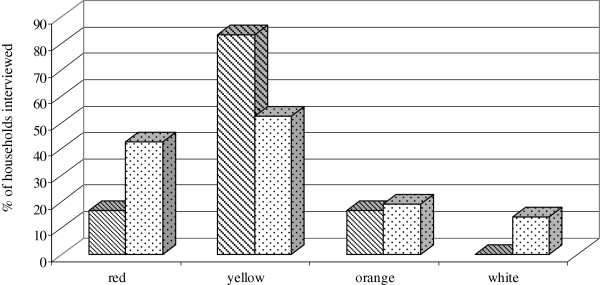
**Preference of varieties bypeople of San Luis Atolotitlán;people of Coatepec.** (Percentage of households interviewed).

As indicated in Figure 2, perception of variation of *S. pruinosus* by local people focuses on fruit types and people’s preferences guide their criteria of artificial selection. People have special preference for sweet flavor (71% of people interviewed) and pulp color (nearly 46% of people interviewed). Preference of pulp color varied among villages. In San Luis Atolotitlán people prefer yellow and red varieties, whereas in Coatepec people prefer yellow varieties over the orange and red varieties (Figure 3). Nearly 24% of all people interviewed said to prefer juicy larger fruits. Few people (8%) said to have special preference for fruits with few or smaller seeds. People distinguish varieties according to their peel thickness (thick and thin) and thorniness (low and high). When asked specifically on these characters nearly 75% of people interviewed said to prefer fruits with thinner peel although some few people (3%) said to prefer fruits with thick peel since these are more durable when stored. Most people (73%) said to prefer fruits with fewer spines, but some people (5%) said to prefer high thorniness since it is favorable for long distance transporting of fruits in baskets.

Artificial selection on variation of *S. pruinosus* is carried out by all people interviewed, mainly in homegardens and agroforestry systems. In homegardens, nearly 50% of people interviewed said to have planted branches of pitaya trees from other homegardens of their village or from other towns. In agroforestry systems, nearly 65% of people interviewed said to deliberately let standing pitaya trees in their parcels; 77% of them said to do it because of their interest for pitaya fruit. Approximately 15% of people interviewed said to do it with the purpose of establishing living fences for corrals or simply because pitaya trees are appreciated and their presence is not inconvenient. Nearly 54% of people interviewed affirmed to propagate branches of the pitaya trees let standing, particularly those with better fruit.

### Patterns of morphological variation

**Figure 4:**
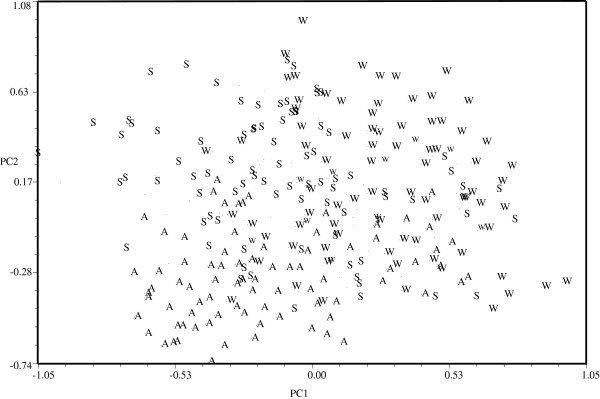
**Projection of individual of *Stenocereus pruinosus *in the space of the first and second principal components (PC).** (W = Wild, S = Silviculturally managed, A = Agriculturally managed).

Multivariate analyses of morphological patterns indicate that in PCA the first three principal components explain nearly 40% of variation. Individual trees managed in different forms showed a gradient of morphological similarity (Figure 4). Most of the agriculturally managed trees are well differentiated from wild and silviculturally managed trees occupying mainly the lower area of the plot, whereas the silviculturally managed and wild trees are not well differentiated among themselves and occupy the middle and upper area of the plot. Eigenvectors show that characters with higher contribution to the first principal component are the dimensions of fruits (higher volume in agriculturally managed than in wild and silviculturally managed populations), total weight (heavier in agriculturally managed than in wild and silviculturally managed populations) and pulp amount (higher in agriculturally managed than in wild and silviculturally managed populations). In the second principal component the most relevant characters were plant size (taller in wild than in silviculturally managed, and intermediate in agriculturally managed populations) and number of branches (more in agriculturally managed than in wild and silviculturally managed populations) (Table 1)

**Figure 5:**
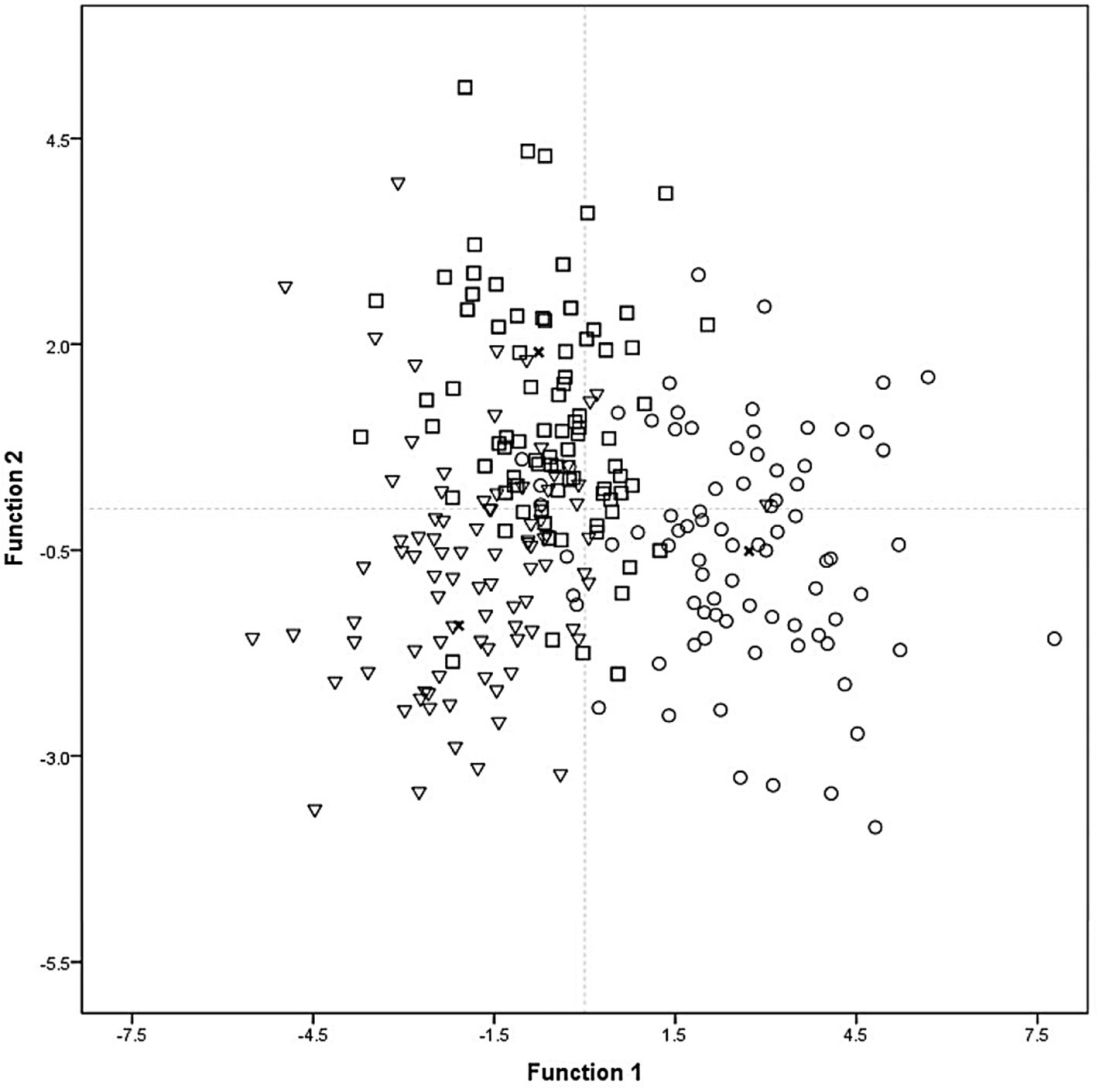
**Classification of *Stenocereus pruinosus *individuals according type of management using Discriminant Function Analysis (DFA).** Δ wild populations; □ silviculturally managed populations, ○ agriculturally managed populations, + centroid group.

**Figure 6:**
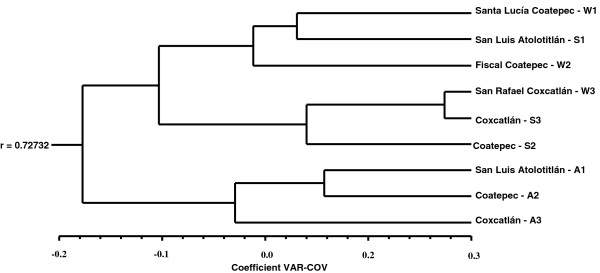
**Classification of *Stenocereus pruinosus *population using Cluster Analysis (CA).** (W = wild; S = Silviculturally managed; A = agriculturally managed).

According to DFA, morphological differences among wild, silviculturally and agriculturally managed trees were significant (Table 4). Most individual trees were classified according to their management type, but a high percentage of wild and silviculturally managed trees are similar among themselves, whereas the agriculturally managed trees are well differentiated (Table 5, Figure 5). The Cluster Analysis (CA) is generally consistent with the results described grouping the populations studied into two main clusters (Figure 6). One of them conformed by wild and silviculturally managed populations and the second one conformed by agriculturally managed populations.

**Table 4:** Significance test of the Multivariate Analysis of Variance (MANOVA)

**Discriminant Function**	**Autovalor**	**% of variance**			**Canonic correlation**
1	4.263				0.9
2	2.006				0.817
Contrast of functions	Wilks’ Lambda	*X*^2^	F	df	significance
1 to 2	0.063	240.23	5.5525	74	<0.000001
2	0.333	95.754		36	<0.000001

**Table 5:** **Classification of wild, silviculturally and agriculturally managed trees of *S. pruinosus *according to the Discriminant Function Analysis (DFA)**

**Actual group**	**Predicted group**					
	**Wild**		**Silviculturally managed**		**Agriculturally managed**	
	**N°**	**%**	**N°**	**%**	**N°**	**%**
Wild	70	77.8	19	21.1	1	1.1
Silviculturally managed	11	13.1	68	81	5	6
Agriculturally managed	4	4.9	8	9.9	69	85.2

#### Morphological diversity and phenotypic differentiation

The total average of morphological diversity of *S. pruinosus* is MD = 0.600, only lower than *Myrtillocactus schenkii* (DM = 0.703) (Table 6). Levels of morphological diversity within the species shows that in silviculturally managed populations (MD = 0.677) was higher than in agriculturally managed populations of homegardens (MD = 0.666), which was in turn higher than in the wild populations (MD = 0.647). However, differences were not statistically significant (among wild and agriculturally managed populations:*X*^*2*^ = 2.14, *df* = 2, *p* = 0.352; among silviculturally managed and wild populations (*X*^*2*^ = 2.08, *df* = 2, *p* = 0.084) (Table 7).

**Table 6:** Comparison between the morphological diversity indexes (MD) and phenotypic differentiation (PD) among populations of six species of columnar cacti from the Tehuacán Valley

**Species**	**Average morphological diversity**	**Phenotypic differentiation wild vs. silviculturally managed populations**	**Phenotypic differentiation wild vs. agriculturally managed populations**
*Escontria chiotilla*	0.550 ± 0.010^4^	0.1549	-
*Polaskia chende*	0.348 ± 0.0461^1^	0.009	-
*Myrtillocactus schenkii*	0.703 ± 0.029^2^	0.069	0.110
*Polaskia chichipe*	0.590 ± 0.0071^1^	0.193	0.353
*Stenocereus stellatus*	0.453 ± 0.0152 ^3^	0.251	0.379
*Stenocereus pruinosus*	0.600 ± 0.009^4^	0.070	0.277

^1^Blancas *et al.*[47], ^2^Blancas *et al.*[46], ^3^Casas *et al.*[32] and ^4^this study.

**Table 7:** **Average of Morphological Diversity (MD) indexes of *Stenocereus pruinosus *populations per management type**

**Populations by type of management**	**Morphological diversity**
Wild Group	0.647 ± 0.021
Silviculturally managed Group	0.677 ± 0.020
Agriculturally managed group	0.666 ± 0.016
Total average	0.600 ± 0.009

Phenotypic differentiation between wild and managed populations (PD = 0.070) was one of the lowest recorded among columnar cacti species hitherto. PD was significantly higher between wild and agriculturally managed populations (PD = 0.2765).

### Spatial movement of *S. pruinosus* propagules: management of gene flow

#### In homegardens

Collecting of branches for planting in homegardens is carried out by 87% of the households interviewed. Most people interviewed said that they collect one to ten branches per year, this practice being more common in Coatepec (79% of households) than in San Luis Atolotitlán (63% of households). Other households interviewed said that they collect ten to twenty branches per year and this practice is more common in San Luis Atolotitlán (33% of the households interviewed) than in Coatepec (13% of the households interviewed). Only 6% of the households interviewed collect 20 to 30 or more branches, and these are households mainly dedicated to produce pitaya for commercialization. One person from Coxcatlán collect and plant annually nearly 120 branches.

In the villages, the branches planted are mainly obtained from other agriculturally managed trees, but according to plants identified in their homegardens by people interviewed, nearly 11% are branches from wild populations. An active interchange of propagules was recorded among nearly 55% of households interviewed, mainly among neighbors of a village (67% of people interviewed interchange pitaya branches), but also among relatives from the town or from other villages (nearly 20% of people interviewed). Branches interchanged are mainly gifts among people and it is uncommon their commercialization.

**Figure 7:**
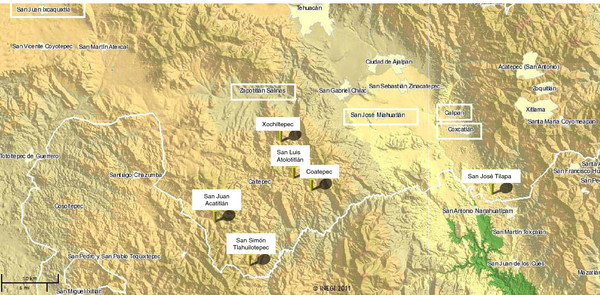
**Location map of the villages mentioned in exchange networks of propagules of *S.pruinosus *in the Tehuacán Valley.** The white boxes indicate the smallest villages in the region, including San Luis Atolotitlán and Coatepec.

Interchange of branches of *S. pruinosus* among villages was recorded on average in nearly 30% of the households interviewed. In San Luis Atolotitlán it is more intense (30%) than in Coatepec (17%). In San Luis Atolotitlán, interchange of branches was documented with the villages of Xochiltepec (7 Km), San Simón Tlahuilotepec (18 Km), San Juan Acatitlán (16 Km), and Zapotitlán (16 Km) (Figure 7). In Coatepec interchange of branches was recorded with the villages of San Luis Atolotitlán (4 Km.) and San José Tilapa (33 Km.). In Coxcatlán, people interviewed said to have interchanged branches of *S. pruinosus* with people from Miahuatlán (14.8 Km), San Luis Atolotitlán (31 Km), San Juan Ixcaquixtla (78 Km) and Calipan (3 Km) (Figure 7).

Protection of seedlings and young plants was recorded not to be a common practice in the Tehuacán Valley. However 15% of the households affirmed that they know this kind of propagation based on personal observations of germination of other plants in natural conditions. In contrast, this practice was referred to by people from the Central Valley of Oaxaca, a neighboring region, wetter than the Tehuacán Valley. There, people collect seedlings and young plants from wild populations and then transplant them to their homegardens (data collected by the authors to be published elsewhere).

#### Introduction of propagules into agroforestry systems

It is common the introduction of branches of trees from homegardens into agroforestry systems, but this practice is much more common in San Luis Atolotitlán (63% of households interviewed) than in Coatepec (37%). In Coxcatlán all households interviewed practice it. Part of the plants occurring in agroforestry systems are let standing but, according to 20% of people interviewed, most plants were already there when they started cultivation of their parcels and supposed that previous people planted them, probably more than one century ago.

## Discussion

### Morphological variation and artificial selection of *S. pruinosus*

Criteria of artificial selection in the Tehuacán Valley may be different among villages and some differences were identified at regional level compared to those reported for La Mixteca region by Luna-Morales *et al.*[51]. In the Tehuacán Valley the main targets of selection are flavor, pulp color, and fruit size, whereas in La Mixteca are particularly relevant pulp color and characters associated with post-harvest manipulation (peel thickness and thorniness). Such differences are likely related to differences in the degree of commercialization of pitaya fruit, which is higher in La Mixteca than in the Tehuacán Valley.

According to the frequencies recorded in interviews, and corroborated with direct questions, in all villages studied and in both regions it is clear the preference of varieties with red and yellow pulp by local people. In La Mixteca such interest is expressed in the recognition and differential management of varieties with seven tonalities of red and eight of yellow pulp [51], whereas in the Tehuacán Valley such interest is expressed in the higher frequencies of these varieties in the managed areas. Tolerance of pitaya trees in agricultural fields is also practiced near the villages and also used for establishing living fences, as observed by the authors in San Mateo del Mar, Oaxaca where the Huave people cultivate pitaya with this purpose rather than to produce fruit (data collected by the authors).

### Consequences of artificial selection on populations of *S. pruinosus*

Our univariate and multivariate morphometric statistical analyses indicate that pitaya trees diverge phenotypically according to management type. In general, phenotypes preferred by people are more abundant in the managed environments and for this reason the average values of some of the characters evaluated differ among population types. The main characters contributing to this pattern are reproductive plant parts, particularly fruit and flower size, and some vegetative parts associated with plant size (plant height and branches number and dimensions). Fruits and flowers size are meaningful for people because of the quality of the main resource provided by the plant, and this fact suggests that artificial selection has favored these features. Although our ethnobotanical interviews did not record artificial selection deliberately favoring robustness of branches, these features may be indirectly selected associated to fruit size. Artificial selection has operated favoring varieties valued by local people and the morphometric study of variation patterns generally confirms ethnobotanical information about use and management. The more frequent phenotypes producing larger fruits with sweeter pulp, thinner peel and less thorniness in agriculturally managed populations (Table 1) illustrate trends in artificial selection similar to those previously documented in *S. pruinosus*[16,51,52] and other columnar cacti species [18,43]. Information derived from these analyses identifies that divergence is higher between wild and agriculturally managed populations than among any other populations; also, that the silviculturally managed populations associated to agroforestry systems are more similar to wild populations (Table 6). This therefore indicates that artificial selection is more significant in homegardens than in agroforestry systems. However, as discussed below, this information is not consistent with that calculated through the phenotypic differentiation index.

Nature of morphological similarities and divergences documented in this study and other columnar cacti remains uncertain [16,18]. Phenotypes are influenced by both genetic and environmental factors and it is particularly relevant to identify the heritability of the characters analyzed in order to identify real evolutionary processes. For the moment it is relevant to say that according to our morphometric studies, some similar phenotypes can be observed in different environments and that variable phenotypes can be observed in similar environments (Table 5). This general observation indicates that morphological features favored by people are not only determined by environmental differences among wild and managed populations but also genetically regulated and therefore, presumably at least partly inherited. Identification of wild phenotypes within agriculturally managed populations is consistent with ethnobotanical information documenting intentional movement of vegetative propagules from wild populations into homegardens and agroforestry systems. Presence of “agriculturally managed phenotypes” in wild populations reveals that these phenotypes naturally occur in the wild but that for some reason they are scarcer there. Hypothetically, since Guillén *et al.*[53] reported differential germination capacity of seeds of wild and agriculturally managed pitaya associated to water availability, their fitness is relatively lower than that of the “wild phenotypes”. This would mean that domestication has artificially favored abundance of “agriculturally managed phenotypes” in artificial environments of homegardens and, in some degree (but markedly lower) in agroforestry systems. Our previous population genetics studies [16,42] reveal that gene flow among all wild, silviculturally and agriculturally managed populations is high. The two processes could be acting simultaneously.

#### Morphological diversity and phenotypic differentiation

Average morphological diversity of *S. pruinosus* is relatively high compared with other species of columnar cacti studied in the Tehuacán Valley (Table 6) [46]. It is only lower than that reported for *Myrtillocactus schenkii*, probably as a consequence of both management intensity and natural adaptations [46]. Although *S. pruinosus* is the cactus species of the Tehuacán Valley under the highest management intensity, it is also the species with higher requirements of moisture for seed germination and establishment [53] which therefore determines higher limitations for sexual reproduction than *M. schenkii*. However, sexual reproduction and seedling establishment is possible throughout time during cyclic episodes of higher rainfall. In addition, the Tehuacán Valley is neighbored by other wetter regions where *S. pruinosus* occur, where presumably sexual reproduction is more frequent and from which plant materials are introduced for cultivation into the Tehuacán Valley.

The high morphological variation documented in *S. pruinosus* could also be a result of the differential selection as a consequence of management and diverse criteria of artificial selection [28]. The spatial movement of branches determined by humans and the highly variable sources of origin of previously selected and cultivated propagules are probably the most important practices that contribute to maintain and increase morphological diversity in agroforestry systems and homegardens, where combined practices (tolerance, promotion, protection and introduction of propagules from homegardens) occur.

Information from the phenotypic differentiation index is not consistent with the multivariate and univariate statistical analyses discussed above. According to this index, phenotypic divergence between wild and managed populations of *S. pruinosus* is one of the lowest reported for all columnar cacti studied in the region (Table 6). Such inconsistency requires still deeper analysis in relation to the method of evaluation itself. We hypothesized that phenotypic divergence was the highest in *S. pruinosus* because it is the most intensively managed species in the region, and this hypothesis is congruent with results from the univariate and multivariate statistical methods used. The pattern identified by this index suggests that although artificial selection is high, the also high gene flow among wild, silviculturally and agriculturally managed populations weaken both morphological differentiation among populations, similarly to that pattern documented for genetic differentiation [16]. Differences in rates of seed germination and seedlings establishment may influence phenotypic divergences within and between species of columnar cacti [53]. Sexual reproduction of *S. pruinosus* is relatively more limited compared with other species because of its relatively high water requirements and it is the species with the most common vegetative propagation [16,42,43]. The relatively low morphological divergence between types of populations is therefore probably due to the high gene flow among populations determined by the spatial movement of vegetative propagules by local people rather than to natural establishment of seedlings. But this is a hypothesis yet to be investigated.

### Gene flow among populations of *S. pruinosus* and human management

The high rate of change in composition of plantations and the continual introduction of branches from the wild and from other towns indicates that artificial selection and gene flow are on-going processes in which human management have high influence. Introduction of branches of *S. pruinosus* from natural populations to homegardens is similar to those practices documented by Casas *et al.*[17] for *S. stellatus* in La Mixteca Baja and Tehuacán Valley, and for *Pachycereus hollianus* by Rodríguez-Arévalo *et al.*[54] in the Zapotitlán Valley. This practice contributes to create genetically rich agroecosystems [16), and its maintenance is crucial for the local and regional conservation of agrobiodiversity [55].

Homegardens are important scenarios for plant experimentation [31], but also important bridges connecting natural populations and other agroforestry systems. Studies of genetics of the populations studied [16] confirm the high level of genetic diversity conserved in this type of agroecosystem [4].

Previous studies document the occurrence of plants that are possible hybrids between *S. pruinosus* and *S. stellatus,* which are maintained in homegardens in la Mixteca and the Tehuacán Valley [17,43,51]. This information makes possible to infer that recruitment of seedlings derived from sexual reproduction has occurred at least in homegardens, probably associated to natural events of high availability of moisture or human supply of water.

Along with the pitaya *S. pruinosus*, other columnar cacti species are managed in homegardens in the villages studied [46,56] where fruit production provides resources to households throughout the year. Agroforestry systems also complement the households’ needs with both agricultural and forest resources [4,41]. Understanding the dynamics of such complementary strategies is therefore necessary for a holistic comprehension of the process of domestication in the context of landscape management. Moreover, the complementarities of different managed spaces where *S. pruinosus* grows and the diversity present on these systems form part of a regional culture of multiple use of natural resources and ecosystems [3,4,57], which is part of a general traditional strategy that looks for the maximization of resources used and minimization of risks [58,59].

Homegardens and milpa agroforestry systems form part of a mosaic of spaces connected because of the movement of propagules by natural means as well as by human actions, from tropical dry forest to homegardens and milpa agrofrorestry systems. This management pattern allows understanding the high genetic diversity and gene flow found in all these systems [16,42], which is the result of the artificial gene flow described along with natural processes determined by the movement of the main pollinators, the bats *Leptonycteris curasoae* and *Choeronycteris mexicana*[60,61] and seed dispersers including several species of birds and bats [4]. It is therefore important to recognize the role of agroecosystems as biological corridors linking natural and artificial populations inside a matrix of environments conforming landscapes [4,62] and its crucial role for biodiversity conservation.

## Conclusions

Management of *Stenocereus pruinosus* in the Tehuacán Valley is associated to a peasant system of subsistence that makes use of multiple resources and ecological units of landscapes, as part of a general strategy that looks for secure plant resources supplying minimizing risks. The system includes use of forests, milpa agroforestry systems and homegardens. The continuous flow of vegetative propagules within and among populations determines important connectivity among management units resembling metapopulations and supports conservation of variation of genetic resources in the Tehuacán Valley [16].

The main targets of artificial selection are fruits flavor and size and secondarily color, peel thickness and thorniness. Needs of traditional peasants contribute to maintain morphological variation mainly directed to direct consumption of fruit rather than commercialization.

The morphometric studies through univariate and multivariate statistical analyses show that plant populations diverge according to management type, suggesting that artificial selection favoring better phenotypes in managed populations is the cause of such pattern. However, the index of phenotypic differentiation tested previously for other columnar cacti species is not consistent with this result, suggesting that high levels of natural and artificial gene flow would be continuously counteracting the consequences of artificial selection and the differentiation of wild from managed populations.

Traditional practices for renewing and moving propagules within and among populations contribute to explain high levels of morphological and genetic variation and low levels of population differentiation in both phenotypic and genetic terms. Networks of propagules exchange between households and villages favor gene flow, high diversity and conservation of agrobiodiversity. Conservation of wild populations and processes of natural and artificial processes of selection and gene flow are key aspects for general conservation of genetic resources.

## Abbreviations

CIECO: Centro de Investigaciones en Ecosistemas Universidad Nacional Autónoma de México; DFA: Discriminant Function Analysis; PCA: Principal Components Analysis; CA: Cluster Analysis; MANOVA: Multivariate Analysis of Variance; MD: Morphological Diversity; PD: Phenotypic differentiation.

## Competing interests

The authors declare that they have no competing interests.

## Authors' contributions

FP conceived, designed and coordinated the study, performed the field survey, carried out the analyses and prepared and drafted the manuscript. JJB participated in statistical analyses and helped to draft the manuscript. AC made substantial contributions to theoretical background, conception and design of the study, field work, data analysis and interpretation of results, and drafted the manuscript. All authors read and approved the final manuscript.

## Authors' information

FP. MSc (Biology) at the Universidad Nacional Autónoma de México (UNAM). Actual a candidate to PhD (Biology) at the Universidad Nacional Autónoma de México (UNAM). JJB MSc (Biology) at the Universidad Nacional Autónoma de México (UNAM). Actual a candidate to PhD (Biology) at the Universidad Nacional Autónoma de México (UNAM). AC. Biologist at UNAM, Mexico, PhD at the University of Reading, UK. Researcher at the Centro de Investigaciones en Ecosistemas, UNAM. Coordinator of the Laboratory of Ecology and Evolution of Plant Resources, advising and conducting researches with ethnoecological, ecological and evolutionary approaches to the study of plant management and domestication.
